# Editorial: Periodontal disease and systemic health: translational insights and clinical approaches

**DOI:** 10.3389/fdmed.2026.1853472

**Published:** 2026-05-20

**Authors:** Deepa Ponnaiyan, Jaideep Mahendra, Anurag Satpathy, Jacqueline Jacinta Dias

**Affiliations:** 1 Department of Periodontology, SRM Dental College Ramapuram, Chennai, India; 2 Department of Periodontology, Meenakshi Academy of Higher Education and Research, Chennai, India; 3 Department of Periodontology, Siksha ‘O’ Anusandhan University, Bhubaneswar, India; 4 Department of Periodontology, Armed Forces Medical College, Pune, India

**Keywords:** biomarkers, cardiovascular diseases, diabetes mellitus, microbiota, periodontitis, systemic inflammation

We would like to present the Research Topic “*Periodontal Disease and Systemic Health—Translational Insights and Clinical Approaches*” highlighting how periodontal disease extends beyond the oral cavity to influence systemic health. The collected articles emphasize emerging translational insights and clinical strategies that bridge oral biology with systemic disease mechanisms. Together, they reinforce the concept of periodontal disease as part of a broader interconnected physiological network rather than an isolated condition. Traditionally viewed as a chronic inflammatory disease of the tooth-supporting structures, periodontal disease is now recognized for its systemic impact. Its pathology involves microbial dysbiosis and host immune responses, resulting in persistent inflammation that can spread beyond the oral cavity through circulating inflammatory mediators and microbial components.

Several articles within this Research Topic explore the mechanistic links between periodontal disease and systemic conditions. A recurring theme is the role of systemic inflammation as a central connecting pathway. Elevated levels of cytokines and acute-phase proteins associated with periodontal inflammation may contribute to endothelial dysfunction, metabolic dysregulation, and immune imbalance. A review article by Xiao et al. emphasizes the role of the oral microbiome in the progression of atherosclerosis. These findings provide a biologically plausible explanation for the associations observed between periodontitis and systemic diseases such as atherosclerosis, cardiovascular disorders and diabetes.

The bidirectional relationship between periodontal disease and metabolic conditions is particularly well emphasized. Hyperglycemic environments are known to exacerbate periodontal tissue destruction through oxidative stress and impaired immune responses. Conversely, periodontal inflammation may negatively impact glycaemic control, suggesting a cyclical interaction that complicates disease management. This has been highlighted in our research topic by Zidane et al. The identification of biomarkers, including inflammatory mediators and metabolic regulators, offers promising opportunities for monitoring disease progression and therapeutic outcomes in both contexts.

Beyond metabolic disorders, the potential link between periodontal disease and neurodegenerative conditions has gained attention. An umbrella review by Arbildo-Vega et al. within this research topic highlights a significant association between periodontal disease and Alzheimer's disease, suggesting that chronic oral inflammation may contribute to neurodegenerative processes, cognitive decline, and neuroinflammation, thereby influencing neurological health through systemic inflammatory pathways and possible microbial dissemination. While causality remains to be definitively established, these findings provide compelling evidence supporting the need for further interdisciplinary research integrating dentistry, neurology, and immunology.

Respiratory health is another domain explored in this collection. Evidence from a systematic umbrella review by Cruzado-Oliva et al. examining the association between periodontal disease and chronic obstructive pulmonary disease suggests that the oral cavity may serve as a reservoir for respiratory pathogens. This supports the hypothesis that periodontal inflammation and oral microbial dysbiosis may influence respiratory disease progression, particularly in susceptible populations, thereby reinforcing the importance of oral health in systemic disease prevention. The oral microbiome emerges as a central theme across multiple studies. Dysbiosis within the periodontal environment is characterized by an imbalance between commensal and pathogenic microorganisms, leading to sustained inflammatory responses. Advances in microbiological and molecular techniques have enabled a deeper understanding of these microbial communities and their functional roles. Ulambayar et al. illustrate, in a nationally representative cohort, an independent correlation between active periodontal disease and peptic ulcer disease, thereby extending the systemic implications of periodontal disease to the gastrointestinal tract. This highlights the gastrointestinal tract as a previously overlooked area of periodontal–systemic interaction and raises significant questions about common microbial and inflammatory mechanisms across mucosal surfaces.

This collection also expands the scope beyond periodontitis to include peri-implantitis, a condition with similar etiology but distinct biological characteristics. Cafferata and Schwarz present evidence that peri-implantitis is associated with elevated systemic inflammatory markers, which may contribute to metabolic dysregulation, renal dysfunction, and neuroinflammatory processes. Crucially, effective peri-implant treatment appears to be capable of lowering circulating inflammatory mediators, whereas implant-supported rehabilitation has been linked to preserved cognitive function and brain volume in older adults. These findings identify peri-implant health as a potentially modifiable determinant of healthy aging, a dimension of oral medicine that has previously received insufficient systemic attention.

Another significant aspect addressed in this Research Topic is the development of diagnostic and predictive tools. In particular, biomarker-based approaches are gaining increasing attention. A systematic review and meta-analysis by Maharavi et al. which investigates Dickkopf-1 as a biomarker highlights its elevated levels in patients with periodontitis and rheumatoid arthritis, suggesting its role in the shared pathogenesis of inflammatory and bone-destructive conditions. Such findings emphasize the potential of molecular markers in improving early diagnosis, risk assessment, and personalized treatment strategies. Furthermore, the integration of biomarkers derived from saliva, serum, and gingival crevicular fluid provides a non-invasive approach to disease detection and monitoring. The application of artificial intelligence for predicting periodontal outcomes represents an important advancement in personalized medicine.

The exploration of immunological pathways also contributes to a deeper understanding of periodontal-systemic interactions. Studies examining immune modulation and inflammatory signalling pathways highlight the complexity of host responses in periodontal disease. Therapeutic strategies targeting these pathways may offer new avenues for managing both oral and systemic conditions, moving beyond traditional mechanical approaches to periodontal therapy. This aspect of immunotherapy has been highlighted in a mini-review by Thanigaimalai et al.

Despite these advances, several challenges remain. The heterogeneity of study designs and patient populations limits the ability to establish definitive causal relationships. Many of the associations described are derived from observational studies, which are subject to confounding factors such as lifestyle, socioeconomic status, and comorbidities. Future research should prioritize longitudinal and interventional studies to determine whether periodontal treatment can significantly alter systemic disease outcomes. From a clinical perspective, the findings presented in this Research Topic emphasize the importance of an integrated approach to healthcare. Collaboration between dental and medical professionals is essential to address the multifaceted nature of periodontal disease and its systemic implications. Routine periodontal evaluation should be considered in patients with systemic diseases, while dental practitioners should incorporate systemic health considerations into their treatment planning.

In addition, the public health implications of periodontal disease cannot be overlooked. Given its high global prevalence, even modest systemic effects may translate into significant healthcare burdens. Preventive strategies, including improved oral hygiene practices, early diagnosis, and increased access to dental care, are critical components of addressing this issue. Education and awareness initiatives targeting healthcare providers and the general population are important in promoting a holistic understanding of health.

In conclusion, this Research Topic provides valuable insights into the complex relationships between periodontal disease and systemic health. The integration of translational research and clinical approaches offers a comprehensive framework for understanding these interactions. As the field continues to evolve, further interdisciplinary collaboration and innovation will be essential to translate these findings into effective clinical practices and improved patient outcomes.

